# Pmel17 Deficiency Affects Melanogenesis and Promotes Tumor Vascularization

**DOI:** 10.3390/ijms27031147

**Published:** 2026-01-23

**Authors:** Justyna Sopel, Katarzyna Sarad, Anna Kozinska, Krystian Mokrzyński, Dariusz Szczygieł, Aleksandra Murzyn, Agnieszka Drzał, Andrzej Słomiński, Małgorzata Szczygieł, Martyna Elas

**Affiliations:** 1 Department of Biophysics and Cancer Biology, Faculty of Biochemistry, Biophysics and Biotechnology, Jagiellonian University, 30-387 Krakow, Poland; justyna.sopel@doctoral.uj.edu.pl (J.S.); anna.kozinska@uj.edu.pl (A.K.); krystian.mokrzynski@uj.edu.pl (K.M.); dariusz.szczygiel@uj.edu.pl (D.S.); aleksandra.murzyn@doctoral.uj.edu.pl (A.M.); agnieszka.drzal@uj.edu.pl (A.D.); martyna.elas@uj.edu.pl (M.E.); 2 Doctoral School of Exact and Natural Sciences, Jagiellonian University, 30-348 Krakow, Poland; katarzyna.sarad@doctoral.uj.edu.pl; 3 Department of Medical Biotechnology, Faculty of Biochemistry, Biophysics and Biotechnology, Jagiellonian University, 30-387 Krakow, Poland; 4 Department of Biophysics, Faculty of Biochemistry, Biophysics and Biotechnology, Jagiellonian University, 30-387 Krakow, Poland; 5 Department of Dermatology, Comprehensive Cancer Center, University of Alabama at Birmingham, Birmingham, AL 35294, USA; aslominski@uabmc.edu; 6 Pathology and Laboratory Medicine Service, VA Medical Center, Birmingham, AL 35294, USA

**Keywords:** melanin, melanosomes, Pmel17, knockout, melanoma, pigmentation

## Abstract

Premelanosomal protein (Pmel, also known as Pmel17) is the major component of melanosomal fibrils and plays a key role in melanin polymerization, making it an important factor in melanogenesis. We investigated how the absence of Pmel affects the properties of B16F10 melanoma cells. Pmel-knockout B16F10 cells were generated using CRISPR/Cas9-mediated genome editing. A viability assay revealed no significant differences between wild-type (WT) and Pmel-knockout (KO) sublines; however, melanosome maturation was impaired. In Pmel KO cells, the cell cycle was disrupted, and higher levels of reactive oxygen species (ROS) were observed compared with WT cells. Moreover, the migration capacity and tube formation of melanoma cells were increased. Tumors derived from Pmel KO cells exhibited unchanged growth kinetics but reduced melanin content, along with enhanced vascularization and oxygenation. Thus, knockout of the Pmel17 gene in melanoma cells alters pigmentation, vascularization, and oxygenation of tumors. These parameters are crucial for both tumor progression and therapeutic response.

## 1. Introduction

Melanoma is a tumor that arises through the malignant transformation of melanocytes—cells of neuroectodermal origin that produce melanin pigment under various regulatory conditions [1,2]. The main role of melanocytes is to produce the biopolymer melanin and, in the skin, to distribute it to epidermal and follicular keratinocytes [3,4,5,6,7]. The primary function of melanin is to protect skin cells from solar ultraviolet radiation and other environmental stressors through diverse mechanisms [8,9,10,11].

There are three main types of melanin found in the human body: black/brown eumelanin, red/yellow pheomelanin, and neuromelanin [4]. Melanogenesis begins with the enzymatic hydroxylation of L-tyrosine to L-DOPA (L-dihydroxyphenylalanine), which is further oxidized to DOPA-quinone, either enzymatically, by tyrosinase, or non-enzymatically [8,12,13]. In the presence of cysteine, pheomelanin is synthesized, while in its absence, eumelanin is produced [5,6]. In addition to serving as precursors for melanin synthesis, L-tyrosine and L-DOPA can regulate the melanogenic machinery and melanocytes functions [14].

Melanin synthesis occurs in highly organized fashion in specialized organelles known as melanosomes [7]. There are four stages of melanosome maturation. In stage I, melanosomes appear as vacuoles—round structures with a clathrin-coated membrane and several internal vesicles (ILVs), which serve as sites for the formation of fibrous structures. During stages I and II, protein fibers elongate and fuse into side sheets. Once fully formed fibrous bands are established, melanin synthesis begins. By stage III, melanin is deposited on these fibrils, which thickens and darkens them. In stage IV, the internal structure becomes completely masked by deposits of melanin [3,8].

Melanosomes containing eumelanin differ in shape from those containing pheomelanin [9,15]. In eumelanosomes, melanin is deposited on a matrix of fibrous sheets composed of amyloid-like fibers formed from the melanocyte protein Pmel17 (also known as ME20M, GP100, silver locus protein homolog, or melanoma-associated antigen ME20) [9]. Pmel17 fibers are responsible for the ovoid shape of melanosomes, which is important for their intracellular transport. Pmel17 is essential for melanin polymerization, condensation, and storage [16]. Although Pmel17 is not required for melanin synthesis itself, it influences melanin accumulation, as seen in animals such as white chickens and silver horses, which carry mutations in the Pmel17 gene. The precise effect of Pmel17 on melanocyte function remains unclear, but it may adsorb oxidative melanin intermediates, thereby preventing damage to the melanosomal membrane [8,10,12].

Factors regulating melanogenesis—such as transcriptional regulators including MITF (microphthalmia-associated transcription factor), or proopiomelanocortin-derived peptides such as MSH (melanocyte stimulating hormone) and ACTH (adrenocorticotropic hormone)—also influence processes related to cell proliferation, epithelial–mesenchymal transition (EMT), invasiveness, and immunosuppression [13,14,17,18,19]. Melanin pigmentation in melanoma cells can become dysregulated, as evidenced by excessive or ectopic accumulation of melanin or its precursors inside or outside the cells [10]. Melanin-mediated photoprotection includes scavenging reactive oxygen and nitrogen species (ROS/RNS). However, ROS/RNS captured by melanin can modify its properties, contributing to mutations that promote cell immortalization and carcinogenesis [17,18,19]. Dysregulated melanogenesis therefore promotes accumulation of mutations in melanoma cells and can alter their metabolic state, influencing melanoma progression [10,15,16]. Additionally, the presence of melanin increases cellular stiffness and reduces elasticity, altering the mechanical properties of melanoma cells [15,20] and potentially diminishes their responsiveness to various therapeutic approaches, thereby affecting disease outcome [10,20,21,22].

In this study, we aimed to investigate how the absence of the major melanosome protein, Pmel17, affects melanoma cells. To this end, we used B16F10 cells, which are capable of producing melanin pigment. Pmel17 expression was knocked out in B16F10 cells using CRISPR/Cas9 gene editing. Two clones lacking Pmel17 expression and two control clones transfected with an empty plasmid were selected. We hypothesized that the loss of Pmel17 would result in decreased melanin content and potentially impair proliferation, migration, and vascular mimicry in melanoma cells. The effects on proliferation were assessed in 2D and 3D cultures, as well as in the CAM (chicken chorioallantoic membrane) and mouse models. Additionally, we evaluated oxygen levels and vascularization in tumors in mice.

Our results show that Pmel17 knockout did not impair growth of B16F10 cells in vitro or in vivo. However, it led to enhanced tumor vascularization and oxygenation. These findings may pave the way for further research into the role of melanosome proteins in melanoma behavior.

## 2. Results

### 2.1. Pmel17 Knockout (KO) Clones

To study the effect of *Pmel17* deficiency on melanoma B16F10 cells, the knockout of the protein-coding gene was generated using the CRISPR/Cas9 method. Cells transfected with the pX459-Pmel17 plasmid were subjected to initial selection using the PCR method (Figure S1A in Supplementary Materials), which resulted in seven clones lacking Pmel17 mRNA and being selected for further analysis. For two of them, C7 and C22, we confirmed lack of expression of the Pmel17 protein (Figure S1B) and lower pigmentation level. These cells are hereafter referred to as Pmel17KO C7 and C22, respectively. Control clones—Pmel17 control, transfected with pX459-empty—were selected to have Pmel17 protein levels similar to the B16F10 cell population (C4, C6).

### 2.2. Pmel17KO Impairs Melanosome Maturation

**Figure 1:**
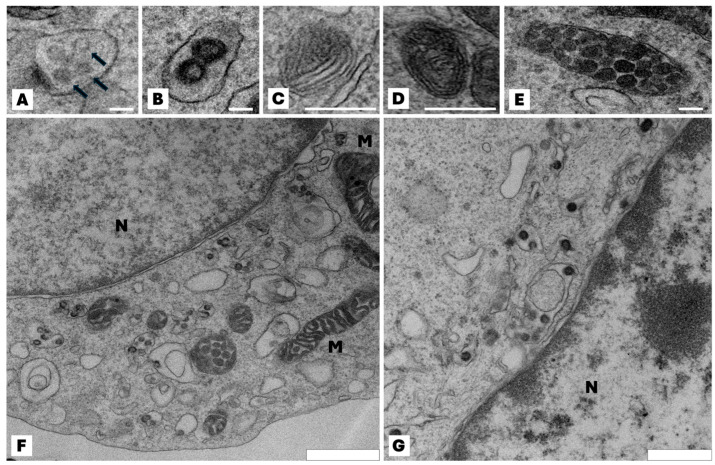
Effect of the lack of Pmel17 protein on the ultrastructural characteristic of B16F10 cells. Representative transmission electron microscopy images of different stages in the development of melanosomes. (**A**,**B**) Multivesicular bodies (arrows) in the coated endosome constitutes the I stage of melanosome development; (**C**) the proteinaceous intralumenal fibrils start appeared—stage III of melanosome development; (**D**) melanin deposit on the fibrils, resulting in their thickening and blackening—stage III of maturation; (**E**) mature melanosome in stage IV. Immature melanosomes (stage I and II) appeared mainly at the Golgi area and mature melanosomes (stage III and IV) in the peripheral area of the cell; (**F**) typical view of B16F10 wt cell; mature melanosomes are visible. All photos (**A**–**F**) are from wild-type B16F10 cells; (**G**) microscopic image of a representative Pmel17 knockout cell (clone C7); stages I and II of melanosome maturation are visible, but no further stages are present in the cell. N—nucleus; M—mitochondria. Scale bar for (**A**–**E**): 100 nm, (**F**): 900 nm, (**G**): 600 nm.

B16F10 wt cells and Pmel knockout have a similar ultrastructural characteristic—they are rounded with some microvilli and have typical nuclei with visible heterochromatin and cytoplasm with mitochondria and other organelles (Figure 1). In B16F10 wt cells eumelanosome formation in four stages was seen. Stages I and II (premelanosomes) do not contain pigment. In stage III polymerization of the fibrillar matrix occurs, resulting in the gradual darkening of the fibrillar sheets and finally masking in stage IV. Mature melanosomes—single membrane-bound organelles containing melanin—were randomly spread in the cell. In Pmel knockout cells the process of melanosome biogenesis stopped at stage II, and subsequent stages of melanosome development were not observed.

### 2.3. Pmel17 KO Affects Melanin Level and ROS Generation

**Figure 2:**
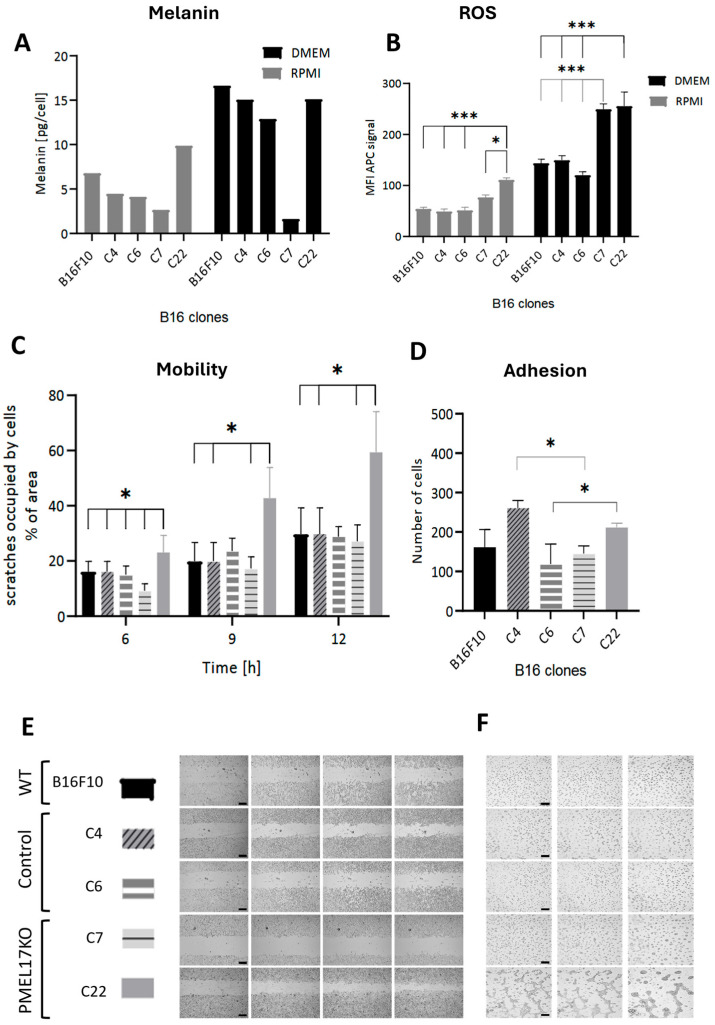
(**A**) Melanin level determined using electron paramagnetic resonance (EPR) technique in B16F10 cells under standard (RPMI) and pigmentation-stimulating (DMEM) conditions. (**B**) Reactive oxygen species (ROS) detection test in living cells, performed in cells in standard culture (RPMI) and stimulated to pigmentation for 2 days (DMEM). Statistical analysis was performed using a one-way ANOVA test, *p* < 0.05 *, *p* < 0.001 ***. (**C**) Effect of the lack of Pmel17 protein on the migration of B16F10 cells; mean values of the percentage of the scratch area occupied by cells after 6, 9, and 12 h relative to time 0. The results are presented as mean ± SD from three experiments, and a two-way ANOVA test was used to calculate the *p*-value: *p* < 0.05 *. (**D**) Effect of the lack of Pmel17 protein on the adhesion of B16F10 cells. Number of B16F10 WT cells and control and Pmel17KO clones adherent to the plate substrate after 1.5 h. The results are presented as mean ± SD of three experiments, and a two-way ANOVA test was used to calculate the *p*-value: *p* < 0.05 *. (**E**) Representative photos of the scratch test at 0, 6, 9, and 12 h after scratching. Scale = 500 μm. (**F**) Effect of Pmel17 knockout on melanoma cell tube formation. Representative photos of pseudotube formation after 4, 6, and 24 h for the B16F10 line, control clones, and Pmel17KO clones. Scale = 200 μm.

The ability of Pmel17KO cells to melanize was analyzed under standard (RPMI) and pigmentation-stimulating (low-glucose DMEM) culture conditions. The melanin concentration in stimulating conditions was 2.5 times higher for B16F10 WT line and approximately 3.4 times higher for Pmel17 control cells (C4, C6). In the Pmel17KO C7 line, it is 40% lower, while in the Pmel17KO C22 line it is 1.5 times higher than control (Figure 2A). These results indicate impaired melanin production/storage.

Pmel KO clones show higher levels of ROS, both under standard and pigmentation-stimulating conditions, compared to WT and Pmel17 controls (C4, C6). This difference is on average 1.7 greater for standard and pigmentation-stimulating conditions (Figure 2B). An increase in ROS levels is observed in all lines under conditions that stimulate pigmentation, but it is significantly higher in Pmel KO clones—approximately two-fold. We propose that defective melanin formation/storage in KO cells enhances the levels of ROS; it is also due to impaired scavenging of ROS because of lower melanin level.

### 2.4. The Effect of Pmel17 Knockout on Cells Adhesion, Migration, and Tube Formation

For the B16F10 line, differences in adhesion between clones were seen (Figure 2D). The largest number of cells adhered to the substrate for the C4 clone. In turn, for Pmel17KO cells, C22 showed the highest adhesion. There was no difference in adhesion between Pmel17KO and WT cells. The wound healing assay showed an increase in migration for Pmel17KO C22 after 9 and 12 h compared to WT and control clones. In contrast, migration of Pmel17KO C7 is reduced compared to WT and control clones after 6 h (Figure 2C,E).

The potential for vascular mimicry of melanoma cells using the ability to form tubes on Geltrex was tested. The WT line, Pmel17 controls (C4, C6), and Pmel17KO C7 line did not form tubes. However, the Pmel17KO C22 line created stable tubes visible already after 4 h of incubation (Figure 2F).

### 2.5. The Effect of Pmel17 Knockout on Cell Proliferation

**Figure 3:**
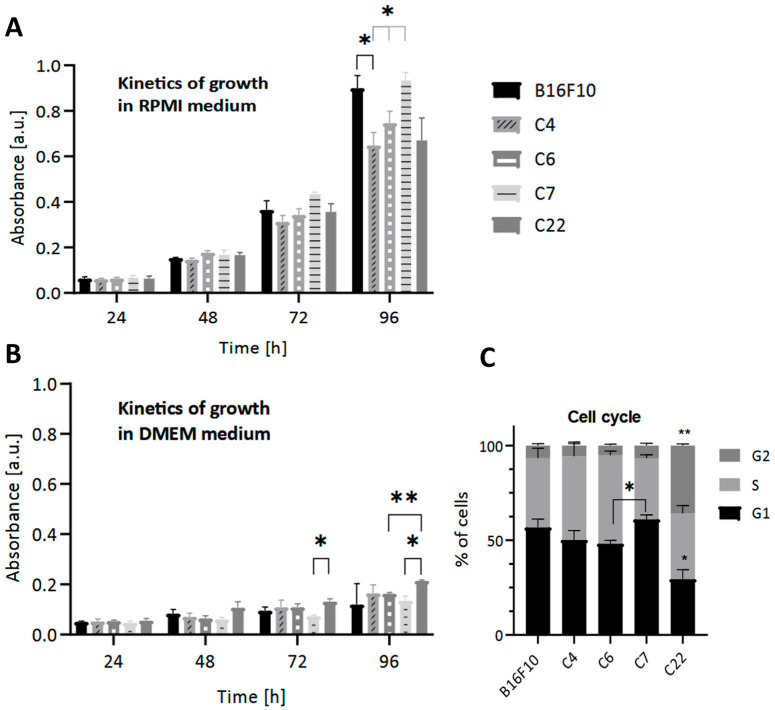
The effect of the lack of Pmel17 protein on the viability of B16F10 cells. The MTT reduction test was performed under (**A**) standard (RPMI) and (**B**) pigmentation-stimulating (DMEM) conditions every 24 h for 4 days. (**C**) The effect of the lack of Pmel17 protein on the cell cycle in B16F10 cells. Clone 4, Clone 6 (control), Clone 7, and Clone 22 (Pmel17KO). The results are presented as a mean ± SD from three independent experiments, and the *p*-value was calculated by 2-way ANOVA, *p* < 0.05 *, *p* < 0.033 **.

As expected, in conditions stimulating pigmentation, we observe a decrease in cell growth compared to low pigmentation conditions in all lines (Figure 3B). Individual clones’ growth rates differed under control conditions. Under pigmentation-stimulating conditions, the values of cell metabolic activity were very similar and much lower than under standard low pigmentation conditions (Figure 3A). Impairment of the pigmentation process correlated with slightly diminished metabolic activity (Figure 3A).

To further analyze the effect of Pmel17KO on proliferation, cell cycle analysis was performed under standard low pigmentation (RPMI) conditions. A higher percentage of cells from the C22 Pmel17KO clone were in the G2 phase compared to the other clones and in the G1 phase compared to WT cells. However, cells of the C7 Pmel17KO clone showed cell cycle arrest in the G1 phase compared to C6 Pmel17 control (Figure 3C).

### 2.6. The Effect of Pmel17 Knockout on Melanin Content, Growth, and Tumor Morphology in CAM Model

**Figure 4:**
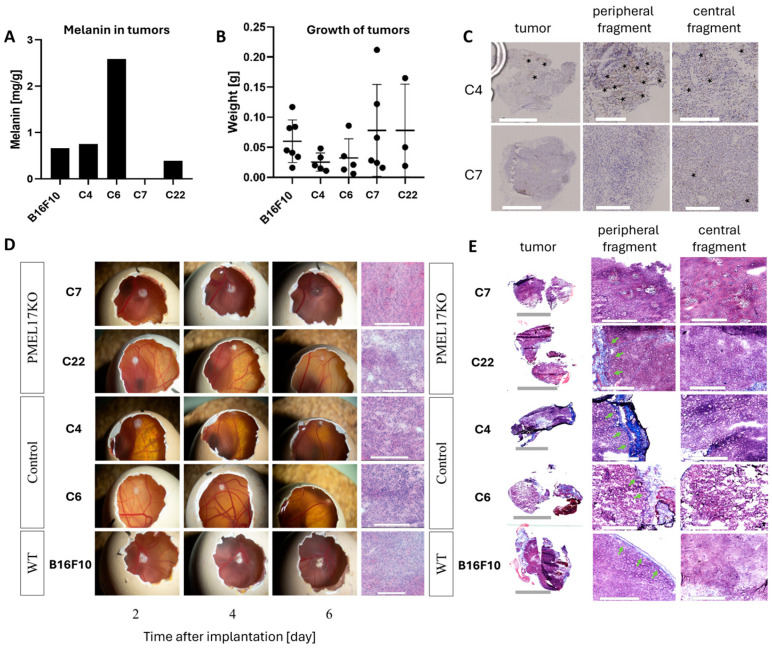
The growth of Pmel17KO tumors on the CAM membrane. (**A**) Melanin level in B16F10 tumors originated from different clones isolated from the CAM model. The EPR signal of melanin was determined per sample mass. (**B**) Weight of tumors isolated from CAM 6 days after melanoma cell implantation. (**C**) Pmel17 staining in tumor tissues obtained from the control (C4) and KO (C7) clones. Asterisks indicate exemplary Pmel17 expression. Scale for peripheral and central fragments = 300 μm. (**D**) Growth and tumor morphology obtained in the CAM model; representative photos 2, 4, and 6 days after implantation of B16F10 cells. Histological preparations visualizing tumor morphology obtained by H&E staining. Scale for tumors in the egg is 5 mm; scale for histological preparations = 300 μm. (**E**) Masson’s trichrome staining, with collagenous material stains in blue, indicated by green arrows. Scale for peripheral and central fragments = 300 μm.

Melanin levels were measured in tumors obtained in the CAM model. The C7 Pmel17KO tumors were amelanotic. The melanin concentration in C22 Pmel17KO tumors is 1.7 times lower than in WT tumors, 1.9 times lower than C4 tumors (Pmel17 control), and 6.6 times lower than C6 tumors (Pmel17 control) (Figure 4A).

All B16 cells were implanted onto the CAM membrane to evaluate tumor growth and morphology. Initially, tumors of the B16F10 line (WT, Pmel17 control, Pmel17KO) grew on the surface of the CAM membrane, then 3–4 days after implantation the tumor began to grow under the membrane (Figure 4D). On 13 EDD tumor weights of Pmel17KO clones showed no differences between Pmel17 control and WT cells (Figure 4B). H&E (hematoxylin and eosin) and Masson’s trichrome staining, however, showed differences in the structure of the tumors. The WT line has a compact structure, without visible areas of necrosis. In the tumors of Pmel17 control clones—C4 and C6—necrotic areas including few areas with hemorrhagic necrosis were visible. The tumor tissue resulting from Pmel17KO clones—C7 and C22—had numerous areas with hemorrhagic necrosis. In C7 tumors it was present throughout the entire tumor, while in C22 tumors necrosis it was seen mostly in the outermost areas of the tumor (Figure 4C). Masson’s trichrome staining did not show intratumoral collagen for any of B16F10 clones. Collagen was only visible within the CAM membrane (Figure 4E).

To evaluate the effect of Pmel17 protein on tumor growth in mice, the C7 Pmel17KO clone and the C4 Pmel17 control were selected. The pigmentation of tumor fragments in the control group varied, while in Pmel17KO tumors it was at a lower level. Immunohistochemistry (IHC) staining for Pmel17 protein shows its expression in very small areas of the tumor, being mostly located at the periphery.

### 2.7. Pmel17 KO Increases Vascularization and Oxygenation of Tumors Growing in a Mouse Model

**Figure 5:**
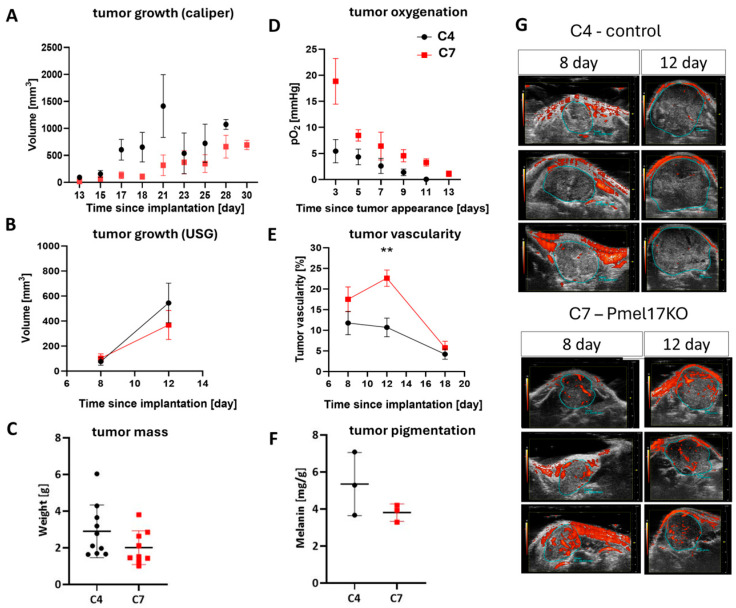
Growth, vascularization, and oxygenation of Pmel17 KO B16F10 tumors in C57Bl/6 mouse model. Control clone—C4 (black circles)—and Pmel17KO clone—C7 (red squares)—were selected for the experiment. (**A**) Growth rate of tumors after implantation of 3000 cells, measured with a caliper from the day of implantation, N = 10 for both groups. (**B**) Tumor growth rate, after implantation of 150,000 cells and analysis of ultrasound images, N = 8 for both groups. (**C**) Tumor mass on the day of isolation, after implantation of 3000 cells. (**D**) Tumor oxygenation level measured by EPR technique with an Oxychip probe localized in tumors growing after implantation of 3000 cells. (**E**) Tumor vascularization after implantation of 150,000 cells and measured by analysis of ultrasound images; for day 12, *p* = 0.0018 **. (**F**) Melanin level determined using EPR signal in isolated tumors per sample mass (18th day of tumor growth after implantation of 150,000 cells). (**G**) Representative ultrasound images performed at 8 and 12 days after implantation of 150,000 melanoma cells. Three tumors from each group are shown. The blue line outlines the tumor, and the red areas within it represent tumor blood vessels with flowing blood.

The analysis of tumor growth, vascularization, and oxygenation was performed for the control clone—C4—and the Pmel17KO clone—C7. To determine the growth and oxygenation of tumors, 3000 cells were injected into an interscapular fat pad. Pmel17KO tumors in this group tended to grow slower than the Pmel17 control tumors, but no statistical differences were detected (Figure 5A,C). Tumors in the control group reached a size qualifying mice for euthanasia slightly faster (the last mice were sacrificed 9 days after tumor appearance, i.e., 28 days after cell implantation) than in the Pmel17KO group, where the last mice were sacrificed 13 days after tumor appearance, i.e., 30 days after cell implantation.

Tumors initiated with 150,000 cells were subjected to ultrasound imaging, during which vascularization was measured. The growth of tumors from these conditions was relatively rapid, and three ultrasound measurements were taken (Figure 5B,E,G). On the 18th day after implantation, the size of the tumors was too high to measure their total volume (Figure 2B), but the mass of very advanced Pmel17KO tumors and control tumors after isolation was compared previously (Figure 5C). No statistical differences in tumor growth were detected between both clones, but Pmel17 KO tumors also tended to grow slower than control tumors (Figure 5B,G). C7 line tumors had higher vascularity than C4 line tumors. After 8 days, C4 tumors had an average of 12% of blood vessels in the tumor volume, and C7 tumors had approximately 18%. On day 12 after implantation, C4 tumors had on average approximately 11% of blood vessels per tumor volume, with C7 tumors having approximately 23% (*p* = 0.0018). On day 18, this difference became insignificant because the percentage volume of blood vessels in both tumor groups decreased to 4% for C4 and 6% for C7 lines (Figure 5E,G).

In line with higher number of functioning blood vessels, the oxygen levels in Pmel17KO tumors were higher than in control tumors from day 3 up to day 11 since tumor appearance (Figure 5D). Together with tumor growth, in both control and Pmel17KO tumors, an overall reduction in oxygen levels was observed. Strong hypoxia with a pO_2_ level below 5 mmHg was reached very quickly in control tumors, while in Pmel17KO tumors such strong hypoxia developed only at a very late stage of tumor growth (Figure 5D).

**Figure 6:**
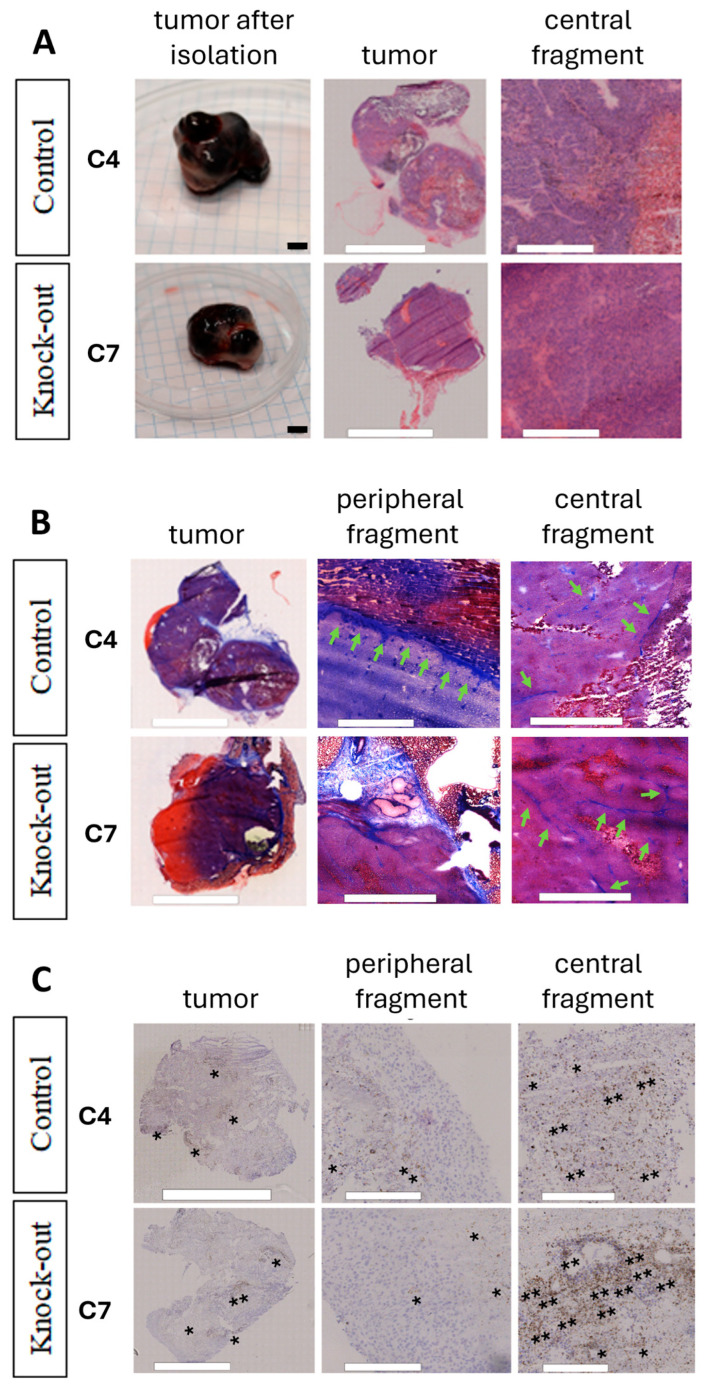
Morphology of Pmel17KO B16F10 tumors growing in C57BL/6 mice. (**A**) Morphology of tumors obtained by H&E. Scale for excised tumors = 5 mm, H&E = 500 μm. (**B**) Masson’s trichrome staining, collagenous material stains in blue, indicated by green arrows. Scale = 300 μm. (**C**) Representative images showing the immunohistochemical staining results for TGF-β. Asterisks indicate exemplary TGF expression. Scale = 300 μm.

In the mouse model, B16F10 tumors also showed necrotic areas. They were visible for both the Pmel17 control line (C4) and Pmel17KO line (C7) (Figure 6A). Additionally, Masson’s trichrome staining showed fibrosis of tissue filled with collagen (Figure 6B). Staining for TGF-β (transforming growth factor β) showed that tumors obtained from C4 Pmel17 control clones had more staining throughout the tumor tissue. In tumors arising from Pmel17KO C7 cells, we also observed the presence of TGF-β throughout the tumor but localized in larger clusters, both in the center and peripheral parts of the tissue (Figure 6C).

## 3. Discussion

### 3.1. Diversity of Clones Obtained Reflects the Heterogeneity of Melanoma Cells

Cancer cells are characterized by high heterogeneity, and different cell clones within a tumor may exhibit distinct properties. B16 melanoma, in particular, is known for its pronounced cellular heterogeneity [23,24,25].

The isolation of two Pmel knockout (KO) clones (C7 and C22) from different B16F10 cell subclones demonstrates that the Pmel KO mutation leads to markedly different behavioral outcomes depending on the cellular background. This suggests that, in melanoma tumors, the same mutation may drive divergent biological behaviors of Pmel KO cells depending on the clonal origin of the tumor cells.

Accordingly, different Pmel KO clones may vary significantly in cell cycle distribution, motility, capacity for vascular tube formation (a phenomenon known as vascular mimicry), ability to form 3D cellular structures, and melanin content in both melanoma cells and tumors (Figure 2, Figure 3 and Figure 4) [26]. The increased potential for tube formation may be due to increased ROS levels [24,27,28,29].

It has been shown that mutations in the same oncogenic pathways may lead to distinct phenotypic outcomes depending on the cellular lineage or mutational background in which they occur [30]. In addition, the clonal heterogeneity and evolutionary history of subclones can modulate their mutagenic potential, even within the same tumor [31,32]. The order and context of preceding mutations further influence the functional consequences of subsequent mutations [33].

In order to make broad, general conclusions regarding the impact of the Pmel17KO mutation on melanoma cell biology, it would of course be necessary to analyze as many Pmel17KO cell clones as possible since their biological properties can sometimes differ substantially in their behavior. Such an approach would allow identification of the widest possible spectrum of changes in melanoma cell biology induced by the Pmel17KO mutation.

It is evident that such a broad spectrum of biological features was not observed in the control clones (C4, C6) to the same extent as in the Pmel17KO clones (C7, C22). Thus, the pronounced differences observed between clones C7 and C22 are most likely the result of the Pmel17KO mutation being superimposed on the pre-existing heterogeneity of the parental B16F10 clones lacking this mutation. The Pmel17KO mutation may therefore lead to a significant increase in cellular heterogeneity within melanoma tumors, and the changes in melanoma cell behavior presented in this study may reflect the spectrum of such potential alterations. 

If an even larger number of Pmel17KO clones were analyzed, an even broader range of phenotypic diversity in melanoma cells induced by the Pmel17 KO mutation would likely be observed as this mutation would be superimposed on an even wider spectrum of heterogeneity present in the parental clones. As is well known, tumor cells exhibit strong heterogeneity; therefore, B16 melanoma cells, which are characterized by high intrinsic heterogeneity, represent a suitable model for such studies.

Nevertheless, the information obtained from only two Pmel17KO clones already provides a substantial insight into the possible changes that may occur in melanoma cells and tumors as a result of this mutation.

Our findings illustrate the heterogeneity of Pmel17KO mutation effects that may potentially occur in melanoma tumors in patients, which are characterized by extensive clonal diversity.

### 3.2. The Influence of Pmel KO Mutation on Melanin Levels in Melanoma Cells and Tumors

Pmel is the key structural protein responsible for the formation of melanin within melanosomes. Mutations in Pmel, including point mutations, deletions, or insertions, lead to hypopigmentation in animals [13]. The absence of Pmel disrupts melanosome architecture, and many melanosomes in *Pmel*−/− melanocytes exhibit dense, granular melanin deposits instead of the smooth, fibrillar structures observed in wild-type cells [34]. In mice with complete Pmel knockout, premature coat graying and moderately reduced coat pigmentation are observed [13]. Hellström et al. further showed that the absence of Pmel does not completely block pigmentation but results in a silvered coat in black mice, decreased pigmentation of paws and tail, and diluted coat color in brown and agouti mice. Quantitative pigment analysis of C57BL/6 *Pmel*−/− mice revealed a 35–69% reduction in eumelanin compared with wild-type animals, whereas pheomelanin levels remained largely unchanged [35].

Pmel17, a key component of melanosome biogenesis, is expressed not only in melanocytes but also in retinal pigment epithelium (RPE), where it ensures the formation of optically dense melanosomes critical for light absorption and oxidative stress protection [36,37,38]. Dysregulation of Pmel17 processing or melanosome maturation in RPE contributes to ocular pigmentation defects and increased retinal susceptibility to oxidative damage, underscoring its importance in both pigmentation and retinal homeostasis [39,40]. Thus, Pmel17 expression in RPE highlights a conserved melanogenic pathway shared with cutaneous melanocytes, reinforcing its role in pigment cell-specific functions. 

In different B16 melanoma cell clones, Pmel gene knockout led to variable reductions in melanin levels. In Pmel KO clone C7 cells, the melanin content in vitro was dramatically reduced compared to control cells (Figure 2). L-tyrosine, a key amino acid in pigmentation regulation, can stimulate MSH-α receptors and promote tyrosinase translocation to melanosomes [41]. However, transferring Pmel KO clone C7 cells from RPMI medium with low L-tyrosine content (0.02883 g/L) to pigmentation-inducing DMEM medium with high L-tyrosine content (0.10379 g/L) did not significantly increase melanin levels, while control cells exhibited a strong melanin increase under high-tyrosine conditions (Figure 2A), similar to physiological conditions in the human body [41].

Furthermore, Pmel KO clone C7 cells formed tumors on the CAM membrane (6 days old) with markedly reduced melanin levels compared to control. In turn, tumors in C57BL/6 mice (18 days old) exhibited only a moderate reduction in melanin content compared with controls (Figure 4 and Figure 5). In contrast, Pmel KO clone C22 cells cultured in vitro displayed no decrease in melanin compared to controls. However, 6-day CAM tumors derived from clone C22 cells exhibited moderately reduced pigmentation compared with control CAM tumors, though this reduction was less pronounced than that observed in clone C7 CAM tumors (Figure 4).

Pmel KO tumors growing on the CAM membrane (6 days old) were characterized by a low melanin content (0.39 mg/g for C22 tumors and 0 mg/g for C7 tumors) and a light coloration (Figure 4A,D), whereas Pmel KO tumors growing in C57BL/6 mice (18 days old) exhibited a high melanin content (3.81 mg/g for C7 tumors) and black pigmentation (Figure 5F and Figure 6A). Such a pronounced difference in the pigmentation of tumors growing on the CAM and in mice is most likely due primarily to differences in tumor growth duration. During 6 days of growth, Pmel KO tumors on the CAM undergo only minimal pigmentation because the time period is too short to allow intensive melanin synthesis. In contrast, 18 days of growth of Pmel KO tumors in mice is sufficient for efficient melanin synthesis and its accumulation in large amounts within the tumor tissue. This is supported by the approximately four-fold lower melanin level (0.98 mg/g) and the light white–gray–red coloration of a Pmel KO tumor growing in a C57BL/6 mouse but isolated at an early growth stage on day 8 after inoculation (Figure S3A–C), among other observations. Thus, the melanin level in this 8-day-old Pmel KO tumor in mice was comparable to the melanin level in 6-day-old tumors growing on the CAM membrane (Figure 4A).

Overall, the Pmel KO mutation produced a wide range of melanin reductions across melanoma clones and experimental conditions. In some cases (e.g., clone C7 in vitro and clone C7 CAM tumors), melanin levels were strongly decreased; in others (e.g., clone C22 CAM tumors and clone C7 mouse tumors), pigmentation was only moderately reduced; and in some contexts (e.g., clone C22 in vitro), melanin accumulation appeared unaffected. These findings indicate that the absence of Pmel protein does not completely abolish melanogenesis but rather impairs it, which is particularly evident in in vitro and CAM models.

Kawahara et al. reported a reduction in stage II melanosomes in SK-MEL-28 *Pmel*−/− cells [28]. Additionally, Chakraborty et al. demonstrated that Pmel accelerates the polymerization of melanin precursors—5,6-dihydroxyindole-2-carboxylic acid and 5,6-dihydroxyindole—into melanins, thereby influencing the efficiency of melanogenesis [42]. Consequently, impaired melanin production and storage in Pmel-deficient cells may contribute to increased susceptibility to UV-induced damage [35].

### 3.3. Increase in ROS Level in Pmel Knockout Cells

Our results show that both Pmel knockout (KO) clones, C7 and C22, exhibit significantly increased levels of reactive oxygen species (ROS) in in vitro cultures compared with control clones. This effect was observed in both RPMI and high-tyrosine DMEM media (Figure 2B). The elevated ROS levels in Pmel KO cells can be explained, at least in part, by reduced melanin content as melanin possesses well-established ROS-scavenging and antioxidant properties through its ability to bind redox-active transition metal ions [18,33,36,42,43,44,45,46].

Another contributing factor may be improper melanin storage within melanosomes in the absence of Pmel protein filaments. Fowler et al. demonstrated that Pmel17 filaments not only provide a structural framework for melanin deposition but also bind highly reactive melanin precursors, thereby preventing cytotoxicity associated with pigment polymerization [38,46,47]. When Pmel is absent, unpolymerized melanin precursors may accumulate and diffuse through the melanosomal membrane into the cytoplasm, where they can be a source of ROS [18,33,36,37].

Additionally, both Pmel KO and control cells exhibited higher ROS levels when cultured in DMEM compared to RPMI (Figure 2B). This may result from the higher glucose concentration in DMEM, which enhances cellular metabolism. Elevated extracellular glucose levels are known to increase oxidative stress and ROS generation through mitochondrial overproduction of superoxide and activation of redox-sensitive signaling pathways [48,49,50,51]. The combination of Pmel gene disruption and high-glucose conditions may therefore synergistically enhance ROS production in melanoma cells, as observed in Pmel KO clones.

Increased ROS levels in Pmel KO cells were already evident in vitro, indicating that this phenotype is independent of angiogenesis. While ROS are known to promote angiogenic signaling in vivo via redox-sensitive signaling pathways such as HIF-1α (hypoxia-inducible factor)/VEGF (vascular endothelial growth factor) [52], their elevation in Pmel-deficient cells most likely reflects impaired melanin-mediated redox buffering rather than a secondary effect of vascular remodeling. Antioxidant modulation might attenuate oxidative stress but would not restore the structural role of Pmel in melanosome organization.

### 3.4. Adhesion and Migration Changes

The scratch assay revealed a reproducible increase in migration (after 6 h, 9 h, and 12 h) of one Pmel KO subline—clone C22—compared with the control and WT lines (Figure 2C,E). Differences between the Pmel KO clones C7 and C22 may result from the pronounced heterogeneity of the B16F10 melanoma cell population. Another potential explanation for the increased migration of clone C22 is the elevated ROS levels observed in these cells compared with clone C7 and the control lines during culture in RPMI medium (Figure 2B).

Endogenous ROS are known to promote cancer cell migration and invasion. Oxidative stress can increase melanoma cell aggressiveness, promote mutation accumulation, and enhance metastatic potential, although excessive ROS may simultaneously reduce cell viability. Moreover, ROS can weaken cell–cell interactions and promote epithelial–mesenchymal transition (EMT) [18]. Supporting this interpretation, we observed a reproducible increase in both migration and ROS levels in B16F10 cells after transferring cultures from low-L-tyrosine to high-L-tyrosine medium.

Melanoma pigmentation may also influence cell migration through the effect of the transcription factor MITF on cell phenotype [51]. Sarna et al. reported that the presence of melanin in SKMEL-188 cells did not alter migration during single-cell tracking; however, this may have been related to the experimental setup. Cells at 90% confluence may display different migratory capacities than those without cell–cell contact. Furthermore, Sarna et al. demonstrated reduced migration of SKMEL-188 cells through 8 μm pore inserts following stimulation of pigmentation [53]. They attributed these differences to the increased stiffness of pigmented cells. In vivo, inoculation of unpigmented SKMEL-188 cells into mice resulted in a higher number of metastases compared with pigmented cells [54]. Similar results were obtained for B16 melanoma cells in mice—unpigmented tumor cells migrated more readily from primary tumors than pigmented ones [55].

Our findings on B16 melanoma cells carrying the Pmel KO mutation are partly consistent with this trend. In some B16 clones, Pmel knockout led to reduced melanin levels and enhanced migration in the scratch assay. While metastasis formation has not yet been assessed in our in vivo experiments, we observed that C57BL/6 mice bearing subcutaneous B16 tumors with the Pmel KO mutation showed a statistically significant increase in lung mass compared with mice bearing control B16 tumors (Figure S2). Subcutaneous B16 tumors typically metastasize to the lungs [56], suggesting that Pmel KO may potentially enhance metastatic spread in B16 melanoma. This hypothesis will be further investigated in our future studies.

We also examined whether pigmentation affects the adhesion of B16 melanoma cells to the substrate. The obtained results did not show a clear effect of the Pmel KO mutation on cell adhesion (Figure 2D). Furthermore, these cells were analyzed for their ability to spontaneously form tubular and 3D structures in the extracellular matrix (Figure 2F). The Pmel KO clone C22 formed elongated cellular aggregates, whereas the WT, control, and C7 Pmel KO clones formed small, spherical aggregates. This difference may reflect altered expression levels of adhesion-related proteins in certain B16 melanoma clones carrying the Pmel KO mutation.

The presence of intracellular melanin has been reported to reduce invasiveness by increasing cell stiffness and reducing elasticity [11,18]. Furthermore, alterations in expression levels of cell adhesion molecules (CAMs) are well documented during melanoma progression and may affect cell–ECM (extracellular matrix) interactions and aggregate morphology [57].

### 3.5. Differences in Growth and Proliferation

Literature data show that pigmented and non-pigmented melanoma cells of the same lineage may differ in their in vitro proliferation rate and in vivo tumor growth rate [58]. Melanin levels may influence the rate of melanoma progression and patient survival [22,58,59,60]. Melanin synthesis itself requires additional energy, which may affect processes related to cell division that also involve numerous biosynthetic reactions and high energy demand. Since the Pmel knockout (Pmel KO) mutation can reduce melanin content in melanoma cells, we hypothesized that it also influences their proliferation rate.

Our results indicate that the Pmel KO mutation in B16 melanoma cells (clones C7 and C22), compared to control cells, did not cause clear or unambiguous changes in in vitro proliferation rate or tumor growth rate in the CAM model (Figure 3A,B and Figure 4B). However, in vivo assessment of subcutaneous B16 melanoma growth in C57BL mice revealed a reproducible trend across independent experiments toward slower growth of Pmel KO clone C7 tumors compared to controls (Figure 5). Slower growth of Pmel KO tumors in mice, observed in 18-day (150,000 cells) and 30-day (3000 cells) experiments, became apparent only at later stages of tumor development. This may explain the absence of detectable growth differences in the CAM model, which allowed only short-term tumor observation over six days. At later stages of B16 tumor growth in mice, changes such as reduced vascularization, decreased oxygenation, increased necrosis, and enhanced pigmentation become particularly evident. These factors could collectively contribute to the slower growth rate of Pmel KO tumors in vivo (Figure 5).

It should also be noted that in vitro analyses revealed statistically significant differences in the duration of specific cell-cycle phases in Pmel KO cells—specifically, an extended G1 phase in clone C7 and shortened G1 but prolonged G2 phase in clone C22, compared to control cells (Figure 3C). Therefore, it cannot be ruled out that alterations in the G1 and G2 phases of the cell cycle in Pmel KO cells may also contribute to the net slowdown in tumor growth [61,62].

Recent studies employing Pmel knockout (Pmel KO) or domain-specific Pmel disruption have provided new insights into how this structural melanosomal protein influences melanoma cell phenotype. Kawahara et al. [28] demonstrated that complete Pmel knockout in melanoma cells disrupts fibril biogenesis and melanosome architecture, confirming the essential structural role of Pmel in pigment granule formation and maturation. Similarly, Hodges et al. [63] used CRISPR/Cas9-mediated deletion of the Pmel repeat domain and observed altered amyloid fibril formation, suggesting that Pmel contributes not only to pigment organization but also to broader aspects of melanoma cell physiology. Although most of these studies primarily focus on melanosome structure and amyloidogenesis, some data indicate that Pmel-related amyloid aggregates can modulate intracellular signaling pathways, including YAP-dependent transcriptional programs associated with cell proliferation and metastasis [64]. Thus, perturbation of Pmel expression may indirectly affect melanoma growth dynamics through alterations in cell signaling and metabolic state.

Taken together, current evidence suggests that Pmel is not only a structural component required for proper melanosome formation but may also influence melanoma cell behavior through indirect mechanisms. Loss or modification of Pmel affects melanosomal maturation, pigment deposition, and amyloid fibril formation, which can, in turn, alter intracellular redox balance, energy expenditure, and signaling pathways linked to proliferation and survival. Consequently, changes in Pmel expression or function could subtly reshape the metabolic and proliferative profile of melanoma cells without producing overt differences in short-term in vitro proliferation assays. The observed tendency toward slower in vivo tumor growth in Pmel KO clones may thus reflect complex interactions between pigmentation status, tumor microenvironment, and cell-cycle regulation, rather than a simple direct effect on proliferation rate [33].

### 3.6. Oxygenation and Vasculature

The results of this study clearly showed that Pmel KO melanoma tumors growing in mice exhibited a significantly higher partial pressure of oxygen (pO_2_) than control tumors (Figure 5E). This increased pO_2_ in Pmel KO tumors was accompanied by markedly stronger vascularization (Figure 5D), a slower tumor growth rate (Figure 5A), and lower melanin levels (Figure 5F). Each of these tumor characteristics can influence oxygen availability within the tumor microenvironment. Enhanced vascularization enables more efficient oxygen delivery, while slower tumor growth is typically associated with a lower proportion of necrotic areas [65], which are characterized by low pO_2_. Conversely, reduced melanin content in melanoma cells or tumors may result in lower oxygen consumption as melanin synthesis consumes oxygen, and active melanogenesis has been shown to reduce cellular respiration [11,65,66].

The process of angiogenesis in melanoma is stimulated by factors such as VEGF, FGF, and TGF-β, among others. TGF-β increases the expression of IL-8, which in turn promotes angiogenesis. Immunostaining of C4 and C7 tumor tissues for TGF-β showed that K4 tumors contain this protein throughout the entire tumor mass, without prominent clusters. In contrast, in C7 tumors, TGF-β occurs in larger clusters localized near necrotic areas (Figure 6C). The level and localization of TGF-β indicate the presence of signals stimulating the angiogenic process. Moreover, HIF-1α induces increased expression of TGF-β, which in turn promotes fibroblast proliferation and ECM synthesis [67]. Additionally, under hypoxic conditions, TGF-β levels increase in cancer cells [68]. Increased oxygenation levels and a lower amount of TGF-β in the obtained PMEL17KO tumors may indicate a more efficient angiogenic process in these tumors, which was also demonstrated in this study.

Differences in pO_2_ levels and vascularity were also observed between early- and late-stage melanoma tumors (Figure 5D,E) and are a general feature of various types of cancer [69,70]. Early-stage tumors displayed significantly higher pO_2_ values (Figure 5E), increased vascularity (Figure 5D), smaller tumor sizes (Figure 5A), and approximately four-fold lower melanin content (Figure S3A), compared to late-stage melanoma tumors. This demonstrates that oxygenation, vascularity, tumor size, and pigmentation are interrelated and may influence one another. It is difficult to clearly indicate which of these parameters plays the dominant role in the increased pO_2_ observed in Pmel KO tumors, in comparison to control tumors. However, stronger vascularization appears to be a key factor, potentially resulting from the slower growth rate of Pmel KO tumors (which allows more time for functional vessel formation) and from their lower melanin content. Melanin may stiffen tumor tissue, impeding angiogenesis, while active melanogenesis imposes additional oxygen demand [11,65,66], which may contribute to local hypoxia and necrosis in more pigmented tumors [71,72]. Enhanced tumor angiogenesis promotes tumor growth, especially in smaller tumors, with the initiation of hypoxia and its signaling. In larger tumors the vicious cycle continues as hypoxia caused by the inefficient vasculature drives further vascular formation. In this context, Pmel17 knockout, resulting in the enhanced tumor vasculature, may promote tumor growth.

### 3.7. Future Directions

The results of this study demonstrated that the Pmel KO mutation influences various parameters of melanoma tumor growth. Building upon these findings, we aim to further investigate the role of the Pmel KO mutation in regulating melanin levels at different stages of melanoma progression as these levels may be critical for the development of hypoxic regions within tumors. Additionally, we plan to assess the effect of the Pmel KO mutation on the concentration of melanin synthesis precursors, which may exert immunosuppressive effects on tumor-infiltrating lymphocytes (TILs). Slominski et al. [73] reported that inhibition of melanogenesis increases the sensitivity of melanoma cells to cytotoxic T lymphocytes activated by interleukin-2 (IL-2), suggesting that reduced melanin synthesis may enhance immune cell-mediated tumor elimination. Of note, oxidate transformation of L-DOPA, a precursor to melanin, showed a dramatic effect of T- and B-lymphocytes activated in vitro [74]. In addition, it has been proposed that inhibition of melanogenesis may decrease the mutational load and change the cellular metabolism [75,76].

Studies using in vitro models have shown that increased cell pigmentation is associated with higher spheroid cells stiffness [77]. Therefore, we intend to assess whether the Pmel KO mutation alters the mechanical properties, including elasticity, of melanoma cells and tumors as tissue mechanics can significantly influence vascularization and metastatic potential.

Because cells of some Pmel KO clones exhibited increased motility and an enhanced ability to form tubes (Figure 2E,F), and mice bearing Pmel KO tumors showed a significantly increased lung mass suggesting the possible development of pulmonary micrometastases (Figure S2B), we plan to investigate in the future whether Pmel KO melanoma cells have an increased metastatic potential in mice.

We aim to evaluate the impact of the Pmel KO mutation on the formation and extent of necrotic regions within tumors. A study by Ma et al. [78] demonstrated that a higher number of CD3^+^ and CD8^+^ TILs in necrotic areas after neoadjuvant anti-PD-1 therapy is associated with a major pathological response and improved 5-year recurrence-free survival in melanoma patients. This suggests that the extent of necrosis and the presence of immune cells within these regions may serve as important prognostic indicators for therapeutic outcomes.

Beyond its established structural role in melanosome biogenesis, Pmel17 has also emerged as a clinically relevant melanoma-associated target. Chen et al. demonstrated that Pmel17 is selectively expressed in melanoma cells and can be effectively targeted using antibody–drug conjugates, resulting in efficient melanoma cell killing in preclinical models [79]. These findings highlight that perturbations in Pmel expression or function may have therapeutic consequences beyond pigmentation itself. In this context, our observations that Pmel knockout alters redox balance, migration behavior, and in vivo tumor growth suggest that targeting Pmel-positive melanoma cells could influence not only cell survival but also broader aspects of tumor biology, including metabolic state and microenvironmental interactions. 

## 4. Materials and Methods

### 4.1. Cell Culture and Pigmentation Induction

The B16F10 murine melanoma cell line was purchased from the American Type Culture Collection (ATCC, Manassas, VA, USA). The authenticity of the line was confirmed by STR profiling (The Genomics Core Facility, Malopolska Centre of Biotechnology of the Jagiellonian University). Cells were cultured in RPMI 1640 medium (Merck Life Science, Darmstadt, Germany) supplemented with 10% fetal bovine serum (FBS; Gibco, Waltham, MA, USA) and penicillin–streptomycin (Merck Life Science, Darmstadt, Germany) at final concentrations of 10 U/mL penicillin and 0.1 mg/mL streptomycin. Cultures were maintained at 37 °C in a humidified atmosphere containing 5% CO_2_.

Pigmentation was induced by replacing the low-tyrosine RPMI medium with low-glucose DMEM, which contains a higher concentration of L-tyrosine (28.83 mg/L in RPMI 1640 vs. 103.79 mg/L in DMEM) [20].

### 4.2. Generation of Pmel17 Knockout Cell Lines Using Cas9-Mediated Genome Editing

Pmel17 knockout (Pmel17KO) B16F10 cells were generated by introducing insertion–deletion mutations into exon 1 of the Pmel17 gene using the CRISPR/Cas9 system. The single guide RNA (sgRNA) was designed using Benchling’s CRISPR design tool (https://www.benchling.com) accessed on 23 November 2020.

The designed sequences (5′-CACCGCACTCAGCACAAGCACGGGA-3′ and 5′-AAACTCCCGTGCTTGTGCTGAGTGC-3′) were phosphorylated, annealed, and cloned into the BbsI-digested pSpCas9(BB)-2A-Puro (PX459) V2.0 plasmid (Addgene #62988) [80]. The resulting construct was designated pX459-Pmel17.

B16F10 cells were transfected with either pX459-Pmel17 or the control pX459 plasmid. After 24 h, transfected cells were treated with 1 µg/mL puromycin for 48 h, then seeded into 96-well plates at a density allowing single-cell growth. Clones were screened for pigmentation potential and Pmel17 mRNA expression using PCR on cDNA synthesized from each clone. cDNA was generated using the High-Capacity cDNA Reverse Transcription Kit (Applied Biosystems™, Carlsbad, CA, USA). PCR amplification was performed with AceQ qPCR Probe Master Mix (Vazyme, Nanjing, China) and the following primers: forward 5′-AGCAACAACCACAGAGGGTC-3′ and reverse 5′-GGCGAGGGAGAAAGAACCAT-3′, following the manufacturer’s protocol.

### 4.3. Western Blotting

Cells were lysed in ice-cold RIPA buffer (Life Technologies, Carlsbad, CA, USA) supplemented with a protease inhibitor cocktail (Roche, Copenhagen, Denmark). Protein concentration was determined using the BCA Protein Assay Kit (Merck Life Science, Darmstadt, Germany). Equal amounts of protein (20 µg per lane) were separated by SDS–PAGE (Tris–glycine system) and transferred onto 0.45 µm PVDF membranes (Life Technologies, Carlsbad, CA, USA). Membranes were blocked with 5% non-fat dry milk in TBST and incubated with rabbit anti-gp100 antibody (1:1000, ab8336-100; Abcam, Cambridge, UK) followed by HRP-conjugated anti-rabbit IgG secondary antibody (1:2000, #7074; Cell Signaling Technology, Danvers, MA, USA). Detection was performed using SuperSignal West Pico PLUS Chemiluminescent Substrate (Life Technologies, Carlsbad, CA, USA) and visualized with a ChemiDoc Imaging System (Bio-Rad, Hercules, CA, USA).

### 4.4. Quantification of Melanin by Electron Paramagnetic Resonance (EPR)

Melanin content in melanoma cells and tumor tissues was quantified using electron paramagnetic resonance (EPR) spectroscopy. The double-integrated EPR signal of samples was compared with that of the synthetic eumelanin model (DOPA-melanin) of a known concentration (1mg/mL) [81] to determine the amount of melanin in the sample and then normalized to cell number or tumor mass [82]. Measurements were performed at pH 7.4 using an EMX-AA EPR spectrometer (Bruker Biospin, Rheinstetten, Germany) operating in the X-band (9.4 GHz) at 77 K.

Biological samples (4 × 10^6^ cells or defined tumor mass) were incubated for 30 min in PBS in a total volume of 200 µL, placed in quartz tubes, and frozen in liquid nitrogen. Before measurement, samples were transferred to a quartz finger-type dewar containing liquid nitrogen and placed in the resonator cavity. Measurement parameters were constant across samples: field center 336.2 mT, sweep width 7 mT, modulation amplitude 0.305 mT, attenuation 38 dB.

### 4.5. Cell Viability Assay (MTT)

Cell metabolic activity under standard (RPMI 1640) and pigmentation-stimulating conditions (low-glucose DMEM) was assessed using the MTT assay. Cell lines were seeded in a 96-well plate at a concentration of 4000 cells per well. Cells were cultured under standard conditions for 24 h. An MTT assay was then performed. Complete RPMI 1640 or DMEM medium containing 0.5 mg/mL MTT (3-(4,5-dimethylthiazol-2-yl)-2,5-diphenyltetrazolium bromide) was added to control and treated wells. After 1 h incubation at 37 °C, the medium was removed, and formazan crystals were dissolved in a 1:1 DMSO/ethanol mixture. Absorbance was measured at 560 nm using a GENios Plus plate reader (Tecan, Mount Waverley, Austria). Measurements were taken every 24 h for 4 days.

### 4.6. Cell Migration (Scratch) Assay

Melanoma cells were cultured in 96-well plates until 90% confluence in RPMI 1640 medium. A uniform scratch was created using a NanoEntek Scratcher (NanoEntek, Seoul, Republic of Korea). Cells were incubated under standard conditions, and wound closure was monitored using an intravital microscope (JuLI™ Stage; NanoEntek, Seoul, Republic of Korea). Images were captured every hour for 12 h at 4× magnification and analyzed in ImageJ version 1.52a (National Institutes of Health, Bethesda, MD, USA).

### 4.7. Cell Adhesion Assay

Melanoma cells were seeded at 500 cells per well in 96-well plates and incubated for 90 min in RPMI 1640 medium. Non-adherent cells were removed by washing twice with PBS. Attached cells were fixed with 70% ethanol and stained with crystal violet. Images were captured using a MOTIC stereomicroscope equipped with a DLM Cam PRO 2MP camera (Delta Optica, Warsaw, Poland). Adherent cells were quantified in ImageJ.

### 4.8. Cell Cycle Analysis

Cells were cultured under standard conditions (RPMI 1640 medium) for 48 h. Cell cycle distribution was analyzed as previously described [80] using the Watson (Pragmatic) model in FlowJo v10.9 software (FlowJo, Ashland, OR, USA).

### 4.9. Reactive Oxygen Species (ROS) Analysis

Cells (5 × 10^4^ per well) were cultured for 48 h in RPMI 1640 or low-glucose DMEM. CellROX™ Deep Red Reagent (Invitrogen, Carlsbad, CA, USA) was added to a final concentration of 5 µM, and cells were incubated for 30 min at 37 °C. After washing with PBS, cells were trypsinized and collected for analysis using a FACSCalibur flow cytometer (BD Biosciences, Milpitas, CA, USA). Data were analyzed in FlowJo v10.9 (FlowJo, Ashland, OR, USA).

### 4.10. Tube Formation Assay

Ninety-six-well plates were coated with 50 µL of Geltrex™ (Life Technologies, Carlsbad, CA, USA) lacking growth factors and incubated at 37 °C for 30 min. Melanoma cells (1 × 10^4^) were seeded in 200 µL RPMI 1640 medium per well. Tube formation was observed using an intravital microscope (JuLI™ Stage; NanoEntek, Seoul, Republic of Korea), and images were taken hourly for 24 h at 4× magnification. Quantitative analysis was performed in ImageJ using the Angiogenesis Analyzer plug-in.

### 4.11. Transmission Electron Microscopy (TEM)

Cultured cells under standard or pigmentation-stimulating conditions for 48 h were collected (1 × 10^6^ cells), centrifuged, and fixed. Pellets were fixed in 2.5% glutaraldehyde (G5882; Merck Life Science, Germany) in 0.1 M cacodylate buffer (C0250; Merck Life Science, Germany) at room temperature for 2 h, followed by postfixation in 1% osmium tetroxide. Samples were dehydrated through a graded ethanol series and embedded in PolyBed 812 epoxy resin at 68 °C (08791-500; Polysciences, Warrington, PA, USA). Ultrathin sections (65–70 nm) were cut on a Reichert Ultracut ultramicrotome (Reichert-Jung, Depew, NY, USA), mounted on 300-mesh copper grids coated with Formvar, and contrasted with uranyl acetate and lead citrate. Observations were performed with a JEOL JEM2100HT transmission electron microscope (Jeol Ltd., Tokyo, Japan) at 80 kV.

### 4.12. Animals

The mice were obtained from an accredited animal breeding facility (Animal Facility in Faculty of Biochemistry, Biophysics and Biotechnology, Jagiellonian University, Krakow, Poland). Experiments were conducted using 3-month-old C57BL/6 female mice in accordance with Directive 2010/63/EU of the European Parliament on the protection of animals used for scientific purposes. Protocols were approved by the 2nd Institutional Animal Care and Use Committee (IACUC) in Kraków, Poland (approval no. 68/2018). Animals were housed in community cages under standard laboratory conditions (12/12h light–dark cycle, humidity 60%, temperature 23 °C) with free access to standard rodent chow—Altromin 1319 FORTI—Breeding Diet (ANIMALAB, Poznań, Poland) and drinking water. Mice were provided with a two-week acclimatization period and one week of handling before experiments began. Control vs. Pmel17KO groups were compared; a total of 16 mice were randomly allocated into two equal experimental groups: control (N = 8) and Pmel17KO (N = 8), where the experimental unit was an individual mouse. Random numbers to allocate to groups were generated using the RAND function in Microsoft Excel. The animals were included in the study if the tumor began to grow (the acceptance of knockout tumor cells may have been reduced). Animals were excluded from the experiment if the tumor did not grow.

Research involving chicken embryos up to embryonic day 13 (EDD13) did not require approval from the Local Ethics Committee. European guidelines (Directive 2010/63/EU) consider early embryos (up to EDD13) as tissue.

### 4.13. Chick Chorioallantoic Membrane (CAM) Assay

The CAM assay was performed as previously described [83]. Fertilized chicken eggs were incubated and rotated for 3 days, after which a 3 mm hole was made in the shell and sealed with Parafilm^®^ (Amcor, Zurich, Switzerland), and the eggs were placed in a static position. On EDD6, the hole was enlarged to ~3 cm and resealed with Parafilm^®^. On EDD7, cells were suspended in Geltrex™ (Life Technologies, USA) at 0.5 × 10^6^ cells per 20 µL and applied to a lacerated CAM area. Eggs were incubated at 37 °C and 60–70% humidity. Tumor growth was monitored for 6 days (until EDD13), after which tumors were excised and weighed. Samples, where the experimental unit was a single tumor, were frozen for histological or EPR analysis.

### 4.14. Murine Tumor Implantation and Growth

Two B16F10 sublines were used: C4 (control) and C7 (Pmel17KO). The interscapular fat pad was selected as the inoculation site, providing easy access for assessing growth kinetics, vascularization, and oxygenation. Cells were injected into the fat pad of C57BL/6 mice—either 1.5 × 10^5^ cells in PBS or 3 × 10^3^ cells in Geltrex suspension. Since the initial experiment using 1.5 × 10^5^ cells indicated very rapid growth kinetics, the number of cells used for inoculation was reduced to slow tumor progression and enable repeated oxygen measurements. The OxyChip probe [84] was implanted in tumors derived from 3 × 10^3^ cells once the volume exceeded 1 mm^3^. EPR-based oxygenation measurements were performed three times per week as previously described [69]. Tumor volume was determined thrice weekly using calipers and calculated as V = π/6 × (a × b × c) [85]. Mice were euthanized when tumors reached ~2 cm^3^. Tumor tissues were processed for histological, immunohistochemical, and EPR analyses, and protein lysates were collected for molecular assays. For tumors derived from 1.5 × 10^5^ cells, ultrasound imaging (Vevo 2100; FujiFilm VisualSonics, Toronto, ON, Canada) was performed once a week to assess tumor volume and vascularity.

### 4.15. Histology

Frozen tumor samples from the CAM and murine models embedded in freezing medium (Epredia™, Kalamazoo, MI, USA) were sectioned at 10 µm using a cryostat and mounted on Polysine™ Adhesion Slides (Epredia™, USA). Sections were fixed in 90% ethanol (2 min) and then placed in decreasing concentrations of ethanol (80%, 70%, 1 min). Slides were rinsed in distilled water and stained with Mayer’s hematoxylin (5 min, Merck Life Science, Germany). Excess staining was washed off under running water for 15 min. Slides were placed in 95% ethanol for 1 min and eosin solution (Merck Life Science, Germany) for 90 s. Slides were then dipped 10 times in decreasing concentrations of ethanol (95%, 80%, 70%). The stained slides were sealed with water-based adhesive (Thermo Scientific™, Waltham, MA, USA). Imaging was performed using a Glissando Desktop Scanner (Objective Imaging Ltd., Cambridge, UK).

### 4.16. Immunohistochemistry

Sections were fixed in 96% ethanol (POCH, Gliwice, Poland) and washed in PBST for 10 min. Immunostaining was performed using monoclonal antibodies against SILV/Pmel17 (27329-1-AP; Proteintech, Rosemont, IL, USA; 1:200), TGF-β (BTX130023; GeneTex, Irvine, CA, USA), and CD31 (776997; Cell Signaling, Danvers, MA, USA; 1:200) according to standard protocols.

### 4.17. Statistical Analysis

All statistical analyses were performed using GraphPad Prism version 5.0 (GraphPad, Boston, MA, USA). The specific tests used are indicated in the figure legends. Depending on experimental design, paired *t*-tests, one-way ANOVA, or two-way ANOVA were applied. Differences were considered statistically significant at *p* < 0.05 and marked as follows: *p* < 0.0332 (*), *p* < 0.0021 (**), and *p* < 0.0002 (***).

## 5. Conclusions

Knockout of the Pmel17 gene in B16 murine melanoma cells disrupted the melanosome maturation process, with only the first two stages (premelanosomes) observed. Later stages of melanosome development were visible only in wild-type cells expressing intact Pmel17 protein. This confirms that melanin packaging in melanoma cells is impaired in the absence of Pmel17, which may influence both the overall melanin content in melanoma cells and the biology of the cells and the solid tumors that arise from them.

The biological effects of Pmel17 knockout in B16 melanoma cells and tumors are pleiotropic and may vary between different clones harboring this mutation. However, the Pmel17 knockout did not significantly affect the proliferation rate of melanoma cells in vitro or the growth rate of the solid tumors they form in vivo. Within various B16 melanoma clones, Pmel17 knockout may lead to diverse cellular changes, including reduced melanin levels, increased reactive oxygen species (ROS), enhanced migratory capacity, and elevated formation of melanoma cell tubes. These observations suggest a potential increase in processes such as vascular mimicry, invasiveness, and ROS resistance in Pmel17-deficient cells.

In vivo, B16 melanoma clones with Pmel17 knockout and reduced melanin content form solid tumors that also exhibit decreased tissue pigmentation. These tumors are characterized by increased blood vessel volume and elevated partial pressure of oxygen (pO_2_), while their overall growth rate remains unchanged. Collectively, these findings indicate that Pmel17 knockout in melanoma cells can significantly alter tumor pigmentation, vascularization, and oxygenation—parameters that may have a critical impact on disease progression and therapeutic efficacy.

## Data Availability

The original contributions presented in this study are included in the article/Supplementary Materials. Further inquiries can be directed to the corresponding author.

## Supplementary Materials

The following supporting information can be downloaded at: https://www.mdpi.com/article/10.3390/ijms27031147/s1.

## Abbreviations

The following abbreviations are used in this manuscript:

CAM	chick embryo chorioallantoic membrane
EDD	chicken embryo development day
EMT	epithelial–mesenchymal transition
Pmel	premelanosomal protein
ROS	reactive oxygen species
RNS	reactive nitrogen species
