# Supplementary material for: Pmel17 Deficiency Affects Melanogenesis and Promotes Tumor Vascularization
Source: Int J Mol Sci. 2026 Jan 23;27(3):1147. doi: 10.3390/ijms27031147 (PMC12897883; doi:10.3390/ijms27031147)
Supplement: Supplementary file 1 [file ijms-27-01147-s001.zip › ijms-4067386-supplementary.pdf]

Supplementary files

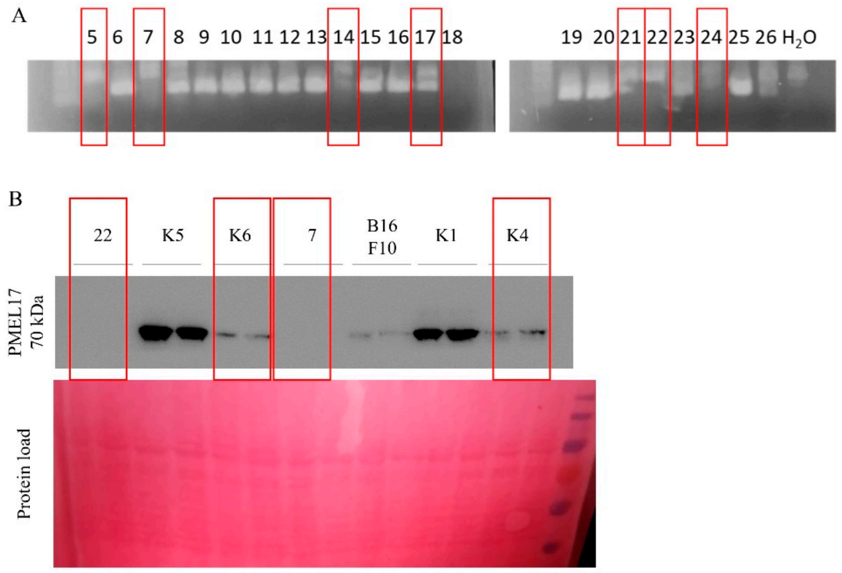

Figure S1. Molecular analysis of clones obtained upon control pX459 and pX459-Pmel17 transduction. (A) The agarose gel electrophoresis of Pmel17 PCR products for each obtained clone. The predicted product size is 163 bp. (B) Western blot analysis of selected cell sublines. Sublines described with numbers were treated with crDNA plasmid, sublines with prefix k are control sublines treated with empty plasmid. Sublines selected for further experiments are marked with a red frame.

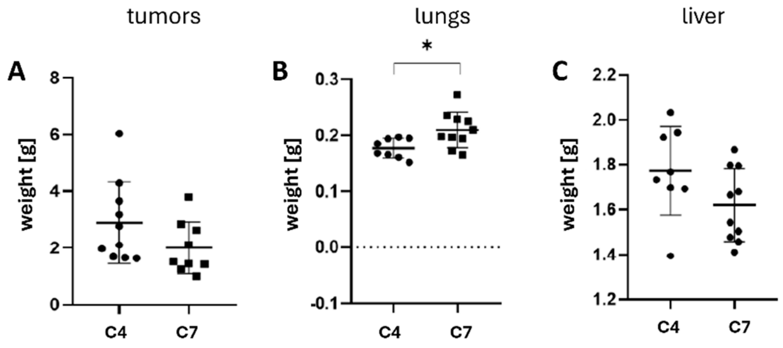

Figure S2. Summary of the weights of A. tumors, B. lungs and C. liver of mice on the day of euthanasia.

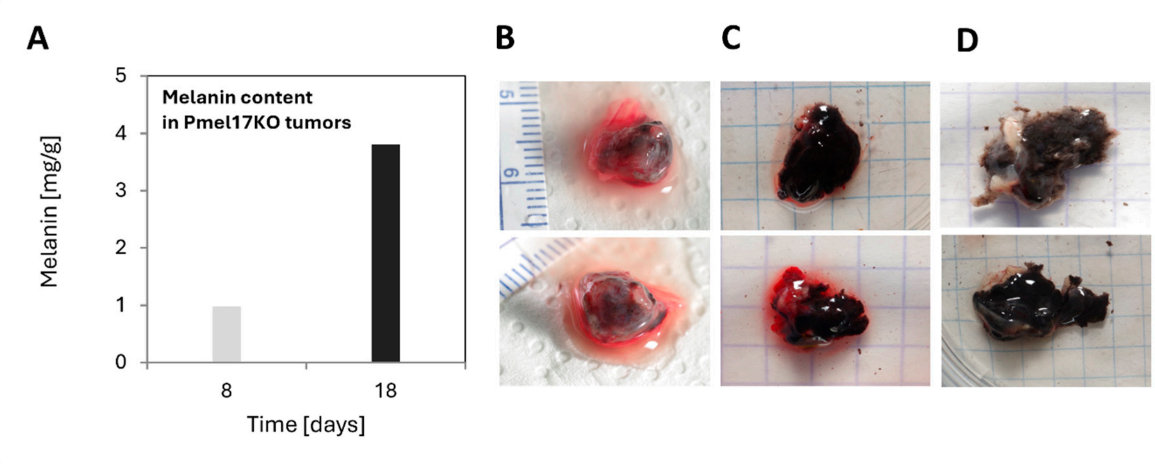

Figure S3. (A) Melanin content in Pmel KO B16F10 tumors (clone 7) growing in C57BL/6 mice, measured on 8th and 18th day of growth using the EPR method. **(B, C)** Pmel KO tumors after isolation on day 8 **(B)** and day 18 **(C)** ; a macroscopically visible higher melanin content is observed at the late stage of the tumors growth. **(D)** Control tumors (clone 4) after isolation on day 18; a macroscopically visible lower blood content in control tumors compared with Pmel KO tumors.
